# Practice and Sociodemographic Factors Influencing Self-Monitoring of Blood Pressure in Ghanaians with Hypertension

**DOI:** 10.1155/2020/6016581

**Published:** 2020-06-05

**Authors:** Kennedy Dodam Konlan, Charles Junior Afam-Adjei, Christian Afam-Adjei, Jennifer Oware, Theresa Akua Appiah, Kennedy Diema Konlan, Jeremiah Bella-Fiamawle

**Affiliations:** ^1^Department of Social & Behavioural Sciences, School of Public Health, University of Ghana, Legon, Accra, Ghana; ^2^Department of Medicine, Nursing Directorate, Korle-Bu Teaching Hospital, Accra, Ghana; ^3^Department of Nursing, St. Karol School of Nursing, Aplaku-Accra, Ghana; ^4^School of Business, Ghana Institute of Management & Public Administration (GIMPA), Accra, Ghana; ^5^Department of Public Health Nursing, School of Nursing and Midwifery, University of Allied Health Sciences, Ho, Ghana

## Abstract

**Background:**

In sub-Saharan Africa, the prevalence of hypertension has assumed epidemic levels and currently accounts for numerous complications such as stroke, heart failure, and kidney damage. Management of hypertension involves both drug and nonpharmacological approaches. Self-monitoring of blood pressure is an important nonpharmacological approach that facilitates early detection of deteriorating blood pressures and complications.

**Aims:**

We determined the practice and sociodemographic factors influencing self-monitoring of blood pressure among Ghanaians with hypertension.

**Methods:**

In a cross-sectional design, we recruited four hundred and forty-seven (447) Ghanaians with hypertension receiving care at the hypertensive Outpatient Department (OPD) Clinics of the Medical Department at the Korle-Bu Teaching Hospital (KBTH). The respondents were sampled using a simple random sampling technique of balloting without replacement. A structured questionnaire was used to gather data on the practice of self-monitoring of blood pressure and sociodemographic factors influencing self-monitoring in the respondents. We also measured some anthropometric and haemodynamic indices of the respondents. The data was entered in Microsoft Excel 2010 and exported into SPSS 21.0 to aid with the data analysis. A chi-square test and Student's *t*-test analysis were done to determine the relationship between the practice of self-monitioring and other sociodemographic variables. Data analayses were conducted at a significant level (alpha 0.05) and power of 95% confidence. Thus, *p* < 0.05 was considered statistically significant.

**Results:**

The practice of self-monitoring of blood pressure was 25.3% with more female respondents claiming to practice self-monitoring as compared to their male counterparts (28.6% vs. 20.7%). Awareness of self-monitoring of blood pressure was associated with increased practice of self-monitoring of blood pressure. Health workers (46.8%), colleague patients (39.8%), relatives/spouses (6.7%), and the media (6.7%) were identified as the sources of information about self-monitoring of blood pressure. Awareness of self-monitoring, level of education, valid health insurance, occupation, income levels, and marital status had a significant relationship with self-monitoring of blood pressure among the respondents. Thus, respondents with higher education, awareness of self-monitoring, valid health insurance, formal employment, and higher income were likely to monitor their blood pressure.

**Conclusion:**

Several sociodemographic factors influence the practice of self-monitoring of blood pressure in Ghanaians with hypertension. Thus, targeted hypertension education and social-cognitive interventions should focus on these sociodemographic factors so as to improve self-monitoring of blood pressure in order to reduce the complications of hypertension.

## 1. Introduction

Hypertension, also known as high blood pressure (BP), is now a global public health problem associated with serious health complications [1]. Hypertension is a leading cause of global burden of heart diseases, stroke, renal failure, peripheral vascular disease, and premature mortality and disability [1, 2]. The global burden of hypertension revealed that 25% of adults are living with hypertension and 9.2% of the total mortality is due to hypertension-related events [3]. It is estimated that over a billion people currently suffer from hypertension [1]. This figure is expected to increase to almost two billion in 2025 [1]. Recent studies from Ghana and other sub-Saharan African countries indicate that the prevalence of cardiovascular diseases (CVDs) is increasing at an alarming rate [3, 4].

The current management of hypertension involves pharmacological and nonpharmacological strategies [4, 5]. The pharmacological approach involves using antihypertensive medications such as beta-blockers, beta-blockers with intrinsic sympathomimetic activity, alpha-1 blockers, combined alpha and beta-blockers, vasodilators, and angiotensin II receptor blocker [5, 6]. Nonpharmacological strategies in the management of hypertension include dietary intake moderation or reduction (particularly saturated fats and excess sodium consumption), regular exercise, and avoidance of exposure to active and passive tobacco smoke [6]. Although significant efforts are being made by healthcare providers to control the morbidity and mortality associated with hypertension, it is still considered a major public health problem [7].

Proper management of hypertension requires adherence to management by patients as well as self-monitoring of blood pressure [8]. Self-monitoring of blood pressure refers to the measuring of one's own blood pressure (BP) outside the usual review visit to a general physician (GP), mostly within one's home [2, 6]. It is the practice where a patient voluntarily measures his/her BP using a self-check device, be it manual or electronic [7, 9]. Self-monitoring of blood pressure is crucial in detecting and preventing hypertension-related complications, and it has been shown to improve patient satisfaction and quality of life and also reduce primary care and out-patient and emergency department visits [10]. Most hypertensive patients in Ghana might not be aware of the enormous role of self-monitoring of blood pressure and hence may not practice efficient self-monitoring of blood pressure [11], thus depriving them of the enormous benefits associated with self-monitoring of blood pressure [9].

## 2. Aim

We determined the practice and sociodemographic factors influencing self-monitoring of blood pressure among Ghanaians with hypertension. In Ghana, complications and mortalities associated with hypertension are still high despite the marked improvement in care [4]. Self-monitoring of blood pressure has been established as one of the effective ways of reducing complications associated with hypertension [10]. Self-monitoring is also useful in getting clients with life-long conditions involved in their management and the surest way of patient empowerment [9, 11].

## 3. Method

### 3.1. Study Design

In this study, we employed a descriptive cross-sectional design. Ghanaians with hypertension receiving care at the Hypertensive Clinic of the Outpatient Department (OPD) of the Medical Department of the Korle-Bu Teaching Hospital (KBTH) were sampled. The philosophical underpinning of the study was the positivist strategy. In adopting the positivist approach to research, which is quantitative, the researcher embarks on the study of the reality by maintaining a distance between him/her and the researcher [12]. Contrary to this approach, researchers who hold the view of interpretivists (qualitative) adopt strategies which will lessen the distance between them and what is being studied [12]. In this study, our view was that self-monitoring of blood pressure and the sociodemographic determinants associated with it can be objectively measured hence the adoption of the positivist approach.

### 3.2. Setting

The study was conducted at the Korle-Bu Teaching Hospital (KBTH), a resource-constrained health facility in Ghana where there is consistent shortage of staff coupled with increasing patient numbers [13]. Thus, empowerment of patients with hypertension through self-monitoring of blood pressure could be useful in enhancing positive disease outcomes through early detection of uncontrolled blood pressures [10]. The KBTH is located in the southern part of Ghana, and it is the national referral hospital in Ghana. It is the third largest hospital in Africa [13]. The KBTH which was established in 1923 has grown from an initial 200 beds to 2000 beds and 17 clinical and diagnostic departments. The hospital is traditionally known to receive huge numbers of referral cases from across the country with daily average patient attendance of 1,500 with average daily admission of 250 patients [13]. The respondents were selected from the Hypertensive OPD Clinics of the Medical Department at the KBTH. These clinics run for four days in a week from 8am to 2pm. The working days for the clinics were Mondays, Tuesdays, Thursdays, and Fridays. The data collection was done on Fridays.

### 3.3. Sample Size Determination

The sample size for the study was determined using the Cochran formula for determining the sample size [14]. Thus, the sample size of 385 respondents was required at a desired precision of 0.05 and at an estimated self-monitoring practice rate of 50%. After adding 20% for nonresponse rate, the sample size for the study was estimated to be 461 respondents.

### 3.4. Selection of Participants and Data Collection

We included male and female hypertensive patients visiting the Hypertensive OPD Clinics of the Medical Department at the KBTH for review post discharge. The inclusion criteria for the selection of the study respondents included Ghanaian hypertensive patients between the ages of 35-65 years, a history of previous admission with hypertension, and being well oriented to time and place with no signs of mental illness.

A simple random sampling technique of balloting without replacement was done using the patients' folders at the Hypertensive OPD Clinics of the Medical Department usually in the morning during waiting time. The patients' folders were numbered, and the corresponding numbers were written on small pieces of paper. The small pieces of paper with the numbers were then placed in a covered container and shaken thoroughly after which thirty (30) papers with corresponding numbers representing patients' folders were selected without replacement. The consent of those selected was obtained, and if they refused to consent, they were replaced by picking other small pieces of paper with the numbers corresponding to specific folders of patients until a total of thirty respondents were selected. The data collection was done on Fridays during a period of fifteen weeks (November, 2018, to March, 2019) excluding public holidays.

A pretested questionnaire was used as the data collection tool. Prior to the data collection, the questionnaire was pretested among hypertensive patients at the Polyclinic of the KBTH. The questionnaire contained sections which asked about the sociodemographic characteristics such as age of respondents, sex of respondents, highest educational level, marital status, occupation, income level, having a valid health insurance, awareness of normal blood pressure, and self-monitoring, as well as the practice of self-monitoring of blood pressure. The data was entered into Microsoft Excel 2010. The data was then exported into the Statistical Package for Social Sciences (SPSS) version 21.0 to aid with the analysis.

### 3.5. Data Analysis

We analyzed the data using the Statistical Package for Social Sciences (SPSS) version 21.0. During the analysis, we did a gender comparison of sociodemographic variables using Pearson's *χ*^2^ test particularly for the dichotomous variables while using Student's *t*-test for a continuous variable (age). In determining the association between self-reported practice of self-monitoring of blood pressure and the sociodemographic variables, we used Pearson's *χ*^2^. In this study, *p* < 0.05 was considered statistically significant.

### 3.6. Ethical Issues

The study data was part of data collected on a protocol titled: Adherence to Management and Quality of Life of Ghanaian Hypertensive on a Structured Health Education Programme (SHEP) approved by the Scientific and Technical Committee and the Institutional Review Board of the Korle-Bu Teaching Hospital (protocol identification number KBTH-IRB/0004/2018). We obtained permission from the Deputy Director of Nursing Service of the Central Outpatient Department prior to data collection. Also, we explained the purpose of the study to each respondent before obtaining written informed consent for the study. Further, we ensured confidentiality of the data collected by assigning unique codes to each response. We also maintained privacy and ensured anonymity throughout the data collection and analysis of the data.

## 4. Results

A total of four hundred and forty-seven (447) respondents took part in the study. As depicted in Table 1, the majority of the respondents (57.9%) were females with 42.1% being males. The mean age of the respondents was 53.13 ± 7.34 years with the females older than the males (54.13 ± 8.46 versus 51.21 ± 4.83). The majority of the respondents (61.3%) were married with only 4.3%, 11.6%, and 22.6% being single, widowed, and divorced, respectively. The results revealed that the majority of the females (44.4%) had no formal education with only 29 (6.5%) of the respondents reporting having had tertiary education. Further, the study found that 46.5% reported as having low income level with 29.97% of the respondents claiming to have high income levels. Female respondents reported high income level as compared to their male counterparts (41.3% versus 14.4%). Also, the results revealed that only 7.4% of the males had valid health insurance as compared to 63.7% of the females. The majority of the respondents did not have valid health insurance. Also, only 10.1% of the males had formal employment and this was similar among the females where 11.6% reported to have formal employment. With regard to unemployment, 41.3% of the females reported to be unemployed as compared to the 25.5% in males. The majority of the males were artisans/technicians or had vocational training (Table 1).

## 5. Awareness and Source of Information about Self-Monitoring of Blood Pressure

The majority (59.3%) of the respondents said they were aware of their normal blood pressure even though most of them could not state the said normal blood pressure. Only, 194 (43.4%) of the respondents said they were aware of self-monitoring of blood pressure with majority claiming not to be aware of self-monitoring. On the sources of information about self-monitoring of blood pressure, 90 (46.4%) claimed health workers, 76 (39.2%) cited colleague patients with only 10 (5.7%), and 18 (9.3%) cited spouses and media houses as sources of self-monitoring information (Table 2).

## 6. Association between Practice of Self-Monitoring of Blood Pressure and Socio-Demographic Factors

The practice of self-monitoring of blood pressure in the respondents was 25.3%. Awareness of self-monitoring of blood pressure was associated with the practice of self-motoring. The study revealed that higher educational qualification was associated with practice of self-monitoring of blood pressure. Respondents with formal employment were likely to self-monitor their blood pressure as compared to their counterparts. Also, having a valid health insurance was associated with the practice of self-monitoring. Further, a high income level was associated with self-monitoring of blood pressure. The results revealed that respondents who were single did more self-monitoring of their blood pressure as compared to married, widowed, and divorced counterparts (Table 3).

## 7. Summary of Results

The results as shown above indicate that the majority of hypertensive patients who seek care at the KBTH were females. Most of the respondents had low or no formal education with the majority under the low income brackets. The results indicate that the awareness of self-monitoring as well as the practice of self-monitoring of blood pressure was low even though awareness of normal blood pressure was reportedly high. The practice of self-monitoring of blood pressure was low and was associated with awareness of self-monitoring, educational level, valid health insurance, income level, and marital status.

## 8. Discussion

A total of 447 respondents were involved in the study, and the majority of the respondents were females indicating that more females sought health care rather than males. This finding is in line with the study done by [14, 15] who found out that more females sought health care as compared to males. On the awareness about self-monitoring, 43.4% of the respondents were aware of self-monitoring with the majority not being aware. Only 25.3% of the respondents claimed to be practicing self-monitoring of blood pressure indicating a low level of practice of self-monitoring. This finding is in line with the study by [6, 16] who found out that only 42% of patients among the respondents were measuring their blood pressure. This finding is in contrast with those found by [11, 15] where 88% of the patients were aware and agreed that self-monitoring of blood pressure was important and actually practiced it.

On the source of information about self-monitoring, health workers served as the main source of information. The media and relatives/spouses as a medium for information on self-monitoring were minimal. This result is in line with the study conducted by [16, 17]. Also, [18, 19] in their study supported the view that the availability of information through family/friend/opinion leaders of trusted groups, the media (including radio, public enlightenment programmes, and newspapers), and the doctor/nurse/health worker can affect one's level of awareness on hypertension. Thus, health workers serve as a key source of information to patients, but the potential role of the media in health promotion is yet to be explored especially in developing countries. None of the respondents said they got their information from the internet despite the increasing internet service availability in Ghana. The need to explore the Internet as a viable means to deliver health educational messages was ought to be emphasized by health providers.

Also, the critical potential of family/spousal support in self-monitoring was found to be low as few of the respondents claimed their family members/spouses helped them with self-monitoring of their blood pressure or even made them aware of it. This finding is in contrast to the findings of [19–21] who observed that family, friends, and churches had powerful influences on patients' long-term management and adoption of healthy lifestyle practice. In a study by [20], 93% of the hypertensive participants reported receiving social support from families while 55% reported receiving social support from friends. Thus, family support remains a crucial need for hypertensive patients if complications of hypertension are to be reduced as they can encourage and support self-monitoring of blood pressure which can eventually reduce the risk of complications.

In our study, we found that the educational level of the respondents was found to be a significant determinant of practice of self-monitoring of blood pressure. The practice of self-monitoring increased marginally with the rising level of education among the respondents. Similar findings have been reported in other studies [16, 20] where the level of formal education was associated with self-monitoring practice in hypertensive patients.

The study found that the practice of self-monitoring of blood pressure increased with increasing self-reported income levels of respondents. Just as found in this study, other studies [16–18] report a positive relationship between the socioeconomic status of patients and the practice of self-monitoring of blood pressure. This could possibly be because of the fact that those within the high income brackets could afford to get blood pressure monitoring devices and thus could practice self-monitoring of their blood pressure at home without any difficulty as compared to their counterparts in the low-income brackets. The respondents in the lower income brackets probably had irregular clinic visits and did not have money to buy the blood pressure gadgets among others hence their low self-monitoring of blood pressure. Similar findings have been reported by some studies which stated that poor attendance to clinics and cost of blood pressure devices were identified by some researchers as a barrier to hypertension self-monitoring [20, 21]. Chronic conditions like hypertension often result in physical disability such as stroke which may decrease one's strength and ability to work. This can reduce the income level of the patient and therefore serve as a source of financial constraint and hence a barrier to hypertensive self-monitoring [15, 17].

The study further found that those who had valid health insurance cards practiced self-monitoring of blood pressure as compared to their counterparts without valid health insurance. It is possible that those with valid health insurance were more likely to be regular in their clinic visitation; hence, they became aware of self-monitoring because it was taught at the OPD by nurses and doctors as compared to their counterparts without valid health insurance. Also, having valid health insurance could reduce the cost of the patient procuring their medications and hence providing alternative income to buy blood pressure devices for self-monitoring. According to [6, 17], resources are needed to support self-monitoring and so a patient's socioeconomic position is of essence when it came to self-monitoring.

## 9. Limitations of the Study

The study used a cross-sectional design making it difficult to infer causation. Also, since the study depended on a pretested questionnaire which largely depended on the respondents' responses and recall, there is high possibility of recall bias. Further, income levels are usually associated with some social status in society; hence, some of the respondents could claim to be in the high social brackets when actually they are not and vice versa.

## Figures and Tables

**Table 1:** Sociodemographic characteristics of respondents.

Variables	Males, *n* (%) 188 (42.1%)	Females, *n* (%) 259 (57.9%)	*χ* ^2^, *p* value
Age (years)	51.21 ± 4.83	54.13 ± 8.46	<0.001^∗^
Marital status, n (%)			87.127, <0.001^∗^
Single	18 (9.6)	1 (0.4)	
Married	90 (47.9)	184 (71)	
Divorced	73 (38.9)	28 (10.8)	
Widowed	7 (3.7)	46 (17.8)	
Highest education, n (%)			135.238, <0.001^∗^
No formal education	90 (47.9)	115 (44.4)	
Junior secondary	38 (20.2)	56 (21.6)	
Senior secondary	22 (11.7)	37 (14.3)	
Vocational/technical	32 (17)	32 (12.4)	
Tertiary	6 (3.2)	19 (7.3)	
Income level, n (%)			74.478, <0.001^∗^
High	27 (14.4)	107 (41.3)	
Middle	29 (15.4)	76 (29.3)	
Low	132 (70.2)	76 (29.3)	
Valid health insurance			143.606, <0.001^∗^
Yes	14 (7.4)	165 (63.7)	
No	174 (92.6)	94 (36.3)	
Occupation			148.080, <0.001^∗^
Formal employment	19 (10.1)	30 (11.6)	
Retired	1 (0.5)	30 (11.6)	
Trading/business	2 (1.1)	60 (23.2)	
Artisan/technician/vocational	118 (62.8)	32 (12.4)	
Unemployed	48 (25.5)	107 (41.3)	

∗ indicates statistical significance (*p* < 0.05).

**Table 2:** Awareness and source of information about self-monitoring of blood pressure.

Variable	Frequency	Percentage
Awareness of normal BP		
Yes	265	59.3
No	182	40.7
Awareness of SM		
Yes	194	43.4
No	253	56.6
Source of SM information		
Media	30	6.7
Health workers	209	46.8
Relatives/spouse	30	6.7
Colleague patients	178	39.8

**Table 3:** Comparing the association between practice of self-monitoring of BP (SM) and sociodemographic factors in the respondents.

Variable	Total, *N* (%)	Practice of SM (%)	*χ* ^2^, *p* value
Awareness of SM			49.240, <0.001^∗^
Awareness of SM	194 (43.4)	81 (41.8)	
Not aware of SM	253 (56.6)	32 (12.6)	
Sex of respondent			3.533, 0.06
Male	188 (42.1)	39 (20.7)	
Female	259 (57.9)	74 (28.6)	
Highest education			253.560, <0.001^∗^
No formal education	205 (45.9)	14 (6.8)	
Junior high school	94 (21.0)	11 (11.7)	
Senior high school	59 (13.2)	8 (13.6)	
Vocational/technical	64 (14.3)	10 (15.6)	
Tertiary	25 (5.6)	8 (32)	
Valid health insurance			32.707, <0.001^∗^
Yes	179 (40)	71 (39.7)	
No	268 (60)	42 (15.7)	
Occupation			256.298, <0.001^∗^
Formal employment	49 (11)	17 (34.7)	
Retired	31 (6.9)	4 (12.9)	
Trading/business	62 (13.9)	8 (12.9)	
Artisan/technician	150 (33.6)	39 (26)	
Unemployed	155 (34.7)	8 (5.2)	
Income level			47.34, <0.001^∗^
Low	208 (46.5)	3 (1.4)	
Middle	105 (23.5)	30 (28.6)	
High	134 (30)	80 (59.7)	
Marital status			45.397, <0.001^∗^
Single	19 (4.3)	14 (73.7)	
Married	274 (61.3)	77 (28.1)	
Divorced	101 (22.6)	6 (5.9)	
Widowed	53 (11.9)	16 (30.2)	

∗ indicates statistical significance (*p* < 0.05).

## Data Availability

The dataset supporting the conclusion of this study is available for systematic review and meta-analysis upon request.

## Abbreviations

SM: Self-monitoring
CVDs: Cardiovascular diseases
BP: Blood pressure.
